# Study of the Population Dynamics and Habitat Suitability of *Oratosquilla oratoria* in the Coastal Waters of Northern Shandong Peninsula, China

**DOI:** 10.3390/ani16172709

**Published:** 2026-09-01

**Authors:** Shengfu Li, Xiaoxia Yu, Xiang Yu, Wenhui Cui, Zhaoyu Song, Min Li, Yiwen Liu, Zhenbo Lv, Liang Zheng, Zhonghua Ren

**Affiliations:** 1 Advanced Institute for Coastal Ecology, Ludong University, Yantai 264025, China; 2023110395@m.ldu.edu.cn (S.L.);; 2 Key Laboratory of Land and Sea Ecological Governance and Systematic Regulation, Ministry of Ecology and Environment, Shandong Academy for Environmental Planning, Jinan 250101, China; 3 Shandong Marine Resource and Environment Research Institute, Yantai 264006, China; 4 Marine and Fishery Integrated Service Center of Haiyang City, Yantai 265100, China

**Keywords:** *Oratosquilla oratoria*, yield-per-recruit (YPR) model, biomass-per-recruit (BPR) model, habitat suitability, spatial management

## Abstract

Fishing pressure shapes fishery-resource populations, but habitat suitability is often overlooked in spatial management. In this study, we investigated the population dynamics and habitat suitability of the mantis shrimp, *Oratosquilla oratoria*, in the northern Shandong Peninsula using surveys from 50 coastal stations in May and August 2024, comprising 6504 individuals. The population exhibited negative allometric growth, with modeled growth accelerating around June. Fishing mortality was 1.44 a^−1^ and the exploitation rate was 0.53. Current F exceeded *F*_0.1_ (0.90 a^−1^) and was close to *F*_20%_ (1.46 a^−1^), while the length at first capture (*L*_c_ = 6.48 cm) was substantially lower than the optimal length at first capture (*L*_copt_ = 10.34 cm), indicating substantial fishing pressure and premature capture of small individuals. GAMs provided better in-sample fits than GLMs. Salinity and water depth were important predictors in both seasons, with nitrite nitrogen retained in spring and total phosphorus and pH retained in summer. Model-derived habitat suitability showed seasonal spatial redistribution, and high-suitability areas experienced greater fishing pressure. We therefore propose a preliminary spatially differentiated management scenario, including reduced fishing effort and candidate *L*_c_ reference values of 11.89 cm and 8.63 cm for high- and moderate-suitability areas, respectively.

## Simple Summary

The mantis shrimp *Oratosquilla oratoria* is an important fishery species in the coastal waters of northern China. Its sustainable use depends not only on fishing pressure but also on the environmental conditions of its habitat. In this study, we surveyed 50 stations in the northern Shandong Peninsula in May and August 2024 to examine the population status and habitat distribution of this species. The results showed that fishing pressure was relatively high and that many individuals were caught before reaching a more suitable capture size. Salinity and water depth were closely associated with shrimp distribution in both seasons, while some other environmental influences differed between May and August. Areas with higher habitat suitability also showed greater fishing pressure. These findings suggest that management should consider both fishing intensity and habitat conditions. Reducing fishing pressure, increasing the size at first capture, and applying different management measures in different habitat areas may improve the long-term use and conservation of this fishery resource.

## 1. Introduction

As crucial interfaces between terrestrial runoff and oceanic environments, the nearshore waters of the northern Shandong Peninsula, located at the confluence of the Yellow and Bohai Seas, sustain traditional fishing grounds, such as Laizhou Bay. Consequently, these nutrient-rich areas function as essential “three grounds and one corridor” habitats, which are pivotal for maintaining the balance of the coastal ecosystem and promoting regional fishery sustainability [1]. However, in recent decades, intense fishing pressure and anthropogenic pollution have severely depleted traditional commercial fishery resources, including the Japanese flounder (*Paralichthys olivaceus*), swimming crab (*Portunus trituberculatus*), and Chinese shrimp (*Fenneropenaeus chinensis*), thereby necessitating stock enhancement and release programs [2,3,4]. Although the mantis shrimp (*Oratosquilla oratoria*) has maintained relatively stable populations owing to its broad ecological niche, it is currently experiencing significant interannual and seasonal alterations in size composition and spatial aggregation [5,6]. Unlike species restored through aquaculture, *O. oratoria* lacks a mature artificial breeding system, rendering its population persistence entirely dependent on natural recruitment. Consequently, when environmental fluctuations or anthropogenic disturbances trigger a population decline, rapid technical recovery remains highly improbable, highlighting the paramount importance of formulating scientifically sound management strategies. Currently, research on *O. oratoria* predominantly focuses on its early life history and spatiotemporal distributions [6,7]. However, its exploitation status under fishing pressure and the associations between seasonal environmental drivers and biomass distribution remain insufficiently understood, particularly in the coastal waters of the northern Shandong Peninsula. Moreover, length-based population dynamics reference points have rarely been integrated with model-derived habitat suitability to determine whether biological and population dynamics characteristics differ among habitat suitability grades. This knowledge gap limits the scientific basis for spatially differentiated regulation of fishing mortality and length at first capture.

Population dynamics serves as the theoretical foundation for elucidating fluctuations in fishery populations and assessing the exploitation status of resources [8]. Traditional models, such as the surplus production model, stock reduction analysis, and catch maximum sustainable yield, often fail to capture the response mechanisms of population structure to fishing selectivity, necessitating targeted models that account for individual biological characteristics [8]. Specifically, the yield-per-recruit (YPR) and biomass-per-recruit (BPR) models can be used to infer exploitation status and recommend optimal ranges for length at first capture [9]. The YPR model helps estimate the biological thresholds required to maximize catch output, whereas the BPR model quantifies changes in total biomass per recruit under different levels of fishing mortality. For example, Yamamoto and Katayama [10] applied the YPR model to the red-spotted grouper (*Epinephelus akaara*), revealing that the current fishing mortality rate (*F*; 0.53) exceeded the maximum fishing mortality rate (*F_max_*; 0.44), suggesting growth overfishing. Similarly, Sun et al. [11] demonstrated using a spawning-biomass-per-recruit analysis that *F* for South Atlantic albacore tuna (*Thunnus alalunga*) must be constrained below 0.55 to maintain a 20% spawning potential ratio and prevent recruitment overfishing. Relying exclusively on a single model presents inherent management limitations; thus, maximizing YPR alone may substantially reduce biomass per recruit, whereas prioritizing BPR may result in neglecting economic yield optimization [12]. Therefore, the combined application of YPR and BPR models facilitates strategies that reconcile ecological security with economic viability [12]. For instance, combined assessments have demonstrated that optimizing the length at first capture (*L*_c_) can help conserve population biomass while maintaining catch yields for species such as the Kabeljou (*Argyrosomus inodorus*) and Japanese eel (*Anguilla japonica*) [9,13].

Beyond fishing pressure, spatial habitat heterogeneity and environmental variations critically impact population dynamics [14,15]. Integrating fishing mortality with habitat suitability is necessary for accurately reflecting the exploitation dynamics of coastal fisheries. Notably, the habitat suitability index (HSI) model helps identify the spatial distribution of potential habitats by quantifying the relationships between species and environment [16]. Selecting appropriate environmental drivers, whether related to extreme climate shifts [7,17,18], nearshore pollution and eutrophication [1], or seasonal and interannual variations [5], is essential for constructing robust HSI models. Furthermore, the selection of model structure is also critical because an inappropriate functional form may distort species–environment relationships and the resulting habitat suitability estimates. The generalized linear model (GLM) provides a parsimonious framework for relatively simple response structures, whereas the generalized additive model (GAM) uses smoothing functions to characterize nonlinear responses without imposing a fixed functional form [19]. A direct comparison of the two frameworks therefore provides an empirical basis for selecting the model that is better supported by the data. Previous comparative studies have highlighted this necessity, identifying GAMs as more suitable for the white-spotted conger (*Conger myriaster*) [19] and GLMs for the South American sea lion (*Otaria flavescens*) [20]. Once selected, the better-supported model can be used to standardize fitted partial effects into a model-derived HSI and stratify the study area into distinct habitat suitability grades. Comparing biological and population dynamics characteristics among these grades can then provide a basis for spatially differentiated management.

In this study, we systematically investigated the exploitation status of the *O. oratoria* resource in the northern Shandong Peninsula, the associations between seasonal environmental drivers and biomass distribution, and differences in biological and population dynamics characteristics among habitat suitability grades. Therefore, we established a comprehensive framework linking population dynamics assessment, habitat suitability evaluation, and spatial management strategy formulation. Specifically, growth equations and length-converted catch curves were utilized to calculate population dynamics parameters. Subsequently, the YPR and BPR models were applied to evaluate current exploitation trends and the responses of yield per recruit and total biomass per recruit to fishing mortality and length at first capture. A comparative analysis of GLM and GAM was conducted to select the better-supported seasonal model for constructing the model-derived HSI and classifying the study area into low-, moderate-, and high-suitability grades. Finally, the integration of habitat suitability with biological and population dynamics characteristics was further used to evaluate spatial variation in exploitation status and provide a basis for the spatially differentiated management of *O. oratoria*.

## 2. Materials and Methods

### 2.1. Sample Collection and Analyses

**Figure 1:**
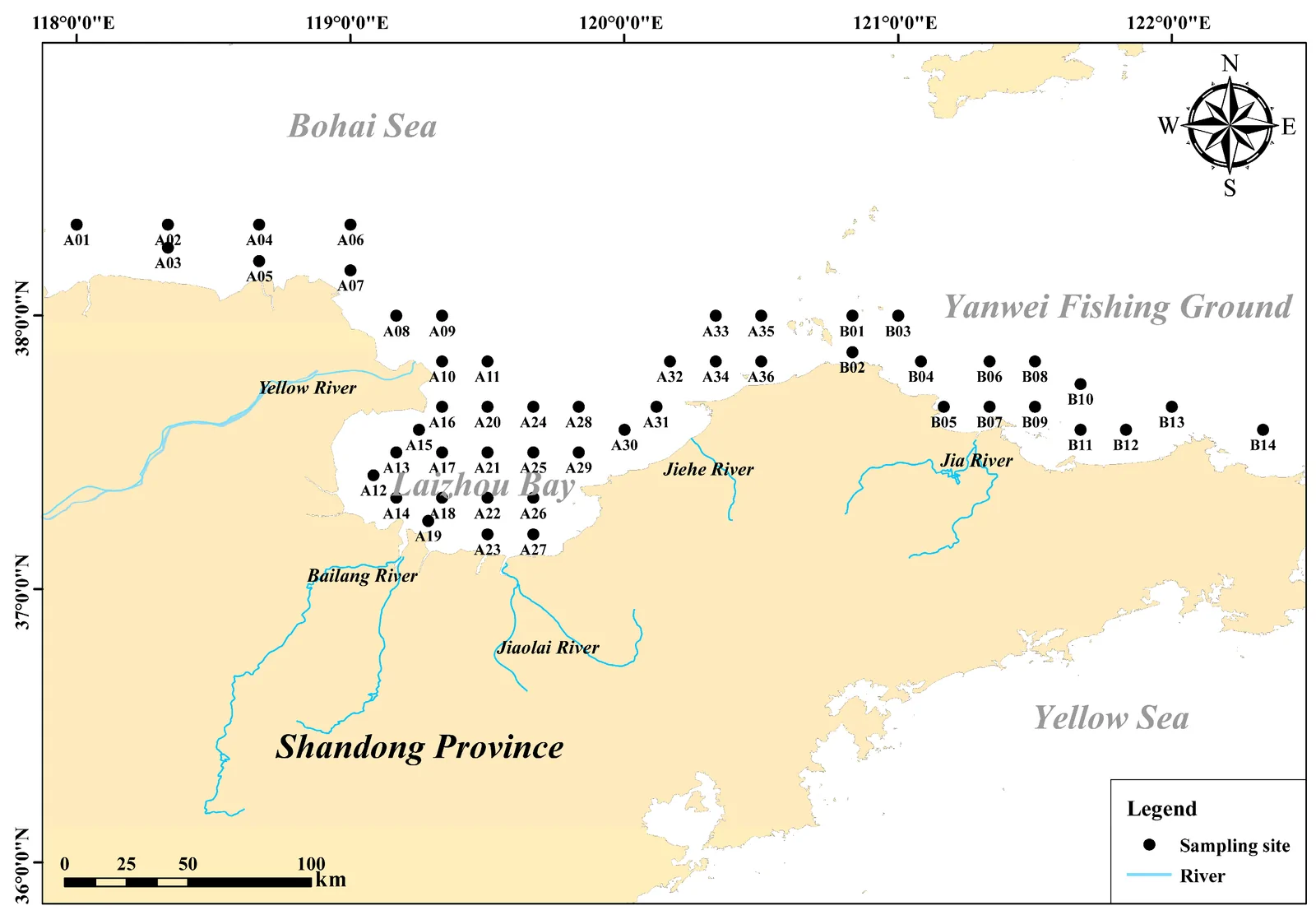
Study area and sampling sites for *Oratosquilla oratoria* and environmental samples in the coastal waters of the northern Shandong Peninsula, China, during spring and summer.

The study area is situated in the nearshore waters of the northern Shandong Peninsula. The same 50 fixed sampling stations were surveyed in May (spring) and August (summer) 2024, yielding 100 station–season tows in total (Figure 1; Table S1). Biological samples were obtained using a chartered fishery survey vessel (260 kW engine power), equipped with a bottom trawl (30.6 m headrope circumference; 20 mm cod-end mesh size). Standardized tows were performed at each station at a speed of 3 knots for 60 min. Upon retrieval, *O. oratoria* individuals were sorted from the catch, rinsed, blotted dry, and immediately frozen. In the laboratory, the body length (*L*) and wet weight (*W*) of these individuals were measured. Biomass and abundance at each station were standardized by the swept area of each tow, which was estimated from towing distance and effective net width, and were expressed as kg·km^−2^ and ind.·km^−2^, respectively.

To evaluate habitat conditions, 16 environmental variables were tracked. A shipborne single-beam echo sounder (Ninglu, Nanjing, China) was used to record water depth (WD). For physicochemical parameters, including temperature (T), salinity (Sal), pH, dissolved oxygen (DO), and chlorophyll *a* (Chl-*a*), we used a portable multiparameter water quality analyzer (ProDSS; YSI, Yellow Springs, OH, USA) to collect in situ measurements. A Niskin water sampler (General Oceanics, Miami, FL, USA) was used to collect seawater from the surface (0.5 m below the water surface), middle, and bottom (1–2 m above the seabed) layers for other chemical analyses. We mixed these layer-specific samples into a single homogenized blend. A 1.0 L subsample was then transferred into a brown high-density polyethylene bottle, cryopreserved, and transported to the laboratory within 24 h. In the laboratory, the levels of dissolved inorganic nutrients, i.e., active phosphate phosphorus (PO_4_^3−^-P), nitrite nitrogen (NO_2_^−^-N), nitrate nitrogen (NO_3_^−^-N), and ammonium nitrogen (NH_4_^+^-N), along with silicate silica (SiO_3_^2−^-Si), were quantified spectrophotometrically. Following digestion, the same method was used to determine total nitrogen (TN) and total phosphorus (TP) concentrations. Additionally, chemical oxygen demand (COD) was determined utilizing the potassium dichromate method, whereas the levels of total suspended solids (TSS) were measured gravimetrically [21], and those of total petroleum hydrocarbons (TPH) were analyzed using ultraviolet fluorescence spectroscopy.

### 2.2. Analysis of Population Dynamics Parameters for O. oratoria

The length–weight relationship of *O. oratoria* was modeled using Equation (1) as follows:

(1) $$W\;=\;aL^{b}$$

where *W* is body weight (g), *L* is body length (cm), *a* is the scaling coefficient of the length-weight relationship, and *b* is the allometric growth coefficient indicating negative (*b* < 3), isometric (*b* = 3), or positive (*b* > 3) allometry [22]. Length-frequency data from 6504 individuals collected in May (*n* = 2814) and August (*n* = 3690) 2024 were pooled for the overall analysis. Individual lengths ranged from 2.1 to 18.0 cm and were grouped into 1.0 cm classes. Habitat-specific analyses included 811, 3394, and 2299 individuals from high, moderate, and low-suitability habitats, respectively. The seasonally oscillating von Bertalanffy growth function (soVBGF) was fitted to the length-frequency data using the ELEFAN algorithm according to Equation (2) [23]:

(2) $$L_{t}\;=\;{\;L}_{\infty }\left[1-e^{-K\left(\;t-t_{0}\right)\;-\left(\frac{\mathrm{KC}}{2\pi }\right)\left(\sin\pi \left(t-t_{s}\right)-\left(\sin2\pi \right)\left(t_{0}-t_{s}\right)\right)}\right]\;,$$

where *L_t_* is the expected body length (cm) at age *t* (a), *L*_∞_ is the asymptotic length (cm), *K* is the growth coefficient (a^−1^), *t*_0_ is the theoretical age at zero length (a), *C* is the dimensionless amplitude of seasonal growth oscillation, and *t_s_* is the fraction of the year marking the start of the seasonal oscillation [23,24].

Using the ELEFAN-derived growth parameters, total mortality (*Z*) was estimated from a length-converted catch curve fitted to eight descending-limb points with length-class midpoints of 10.5–17.5 cm [25,26]. Natural mortality (*M*) was calculated using Pauly’s empirical equation with the observed mean bottom-water temperature of 23.49 °C [27], and fishing mortality and exploitation rate were calculated as *F* = *Z* − *M* and *E* = *F/Z*, respectively. For length classes excluded from the descending limb of the catch curve, dividing the observed catches by expected catches yielded the gear selectivity (*S_j_*). We then fitted a logistic curve to these data points to find the length at first capture (*L*_c_), which represents a 50% capture probability and can be expressed using Equation (3) as follows:

(3) $$S_{j\;}\;=\;\frac{1}{1\;+\;e^{-\;r(L_{j}\;-\;L_{c})}}$$

where *L_j_* is the body length, and *r* is a fitting constant.

These estimated parameters yielded two critical reference points for *O. oratoria* [28]. The optimal length (*L*_opt_; Equation (4)) maximizes cohort biomass. The optimal length at first capture (*L*_copt_; Equation (5)) balances high population biomass with sustainable catch yields. Both *L*_opt_ and *L*_copt_ can be expressed using Equations (4) and (5), respectively, as follows:

(4) $$L_{\mathrm{opt}}=L_{\infty }\;\times \;\frac{3}{\left(3\;+\;\frac{M}{K}\right)}\;,\;\mathrm{and}$$

(5) $$L_{\mathrm{copt}}=\frac{L_{\infty }\left(2+\frac{3F}{M}\right)}{\left(1+\frac{F}{M}\right)\left(3+\frac{M}{K}\right)}$$

### 2.3. Modeling O. oratoria Population Dynamics

*Oratosquilla oratoria* lacks permanent hard structures for age determination [29]. Instead, length-based YPR and BPR models (Equations (6) and (7), respectively) were used to evaluate its population dynamics. These models directly incorporate fishing selectivity and quantify yield and total biomass per recruit and can be expressed using Equations (6) and (7) as follows [30]:

(6) $$\begin{array}{c} \mathrm{YPR}\;\;=\;\sum_{j\;=\;1}^{n}\left[\frac{W_{j}S_{j}F}{S_{j}F\;+\;M}\left(1-\;e^{-(S_{j}F+M)\Delta T_{j}}\right)\exp\left(-\sum_{k\;=\;1}^{j\;-\;1}(S_{k}F\;+\;M)\Delta T_{k}\right)\right] \end{array},\;\mathrm{and}$$

(7) $$\begin{array}{c} \mathrm{BPR}\;=\sum_{j=1}^{n}\left[W_{j}\exp\left(-\sum_{k=1}^{\;j-1}(S_{k}F+M)\Delta T_{k}\right)\Delta T_{j}\right] \end{array},$$

where *W_j_* is the mean weight of the *j*-th length class, Δ*T_j_* is the growth time from *L_j_* to *L_j_*_+1_, and Δ*T_k_* is the duration in the *k*-th length class. The yield per recruit (*Y/R*) and biomass per recruit (*B/R*) were calculated to generate response curves against fishing mortality (*F*). These curves define key YPR reference points: the maximum *Y/R* (*YPR*_max_) at *F_max_*, and the marginal yield threshold (*YPR*_0.1_) at *F*_0.1_. Similarly, the BPR model helps identify the fishing mortalities (*F*_20%_ and *F*_40%_) where *B/R* drops to 20% and 40% of unexploited levels (*F* = 0), setting the biomass reference points *BPR*_0.2_ and *BPR*_0.4_.

Numerical stability was assessed using 30 independent ELEFAN runs with different random seeds while retaining identical length-frequency data, data-processing procedures, parameter bounds, and optimization settings [31]. The resulting parameter variation was propagated through the mortality, selectivity, and per-recruit calculations and was interpreted as algorithmic stability rather than formal bootstrap uncertainty.

### 2.4. Screening of Environmental Factors

In both spring and summer surveys, T, Sal, WD, pH, and the levels of DO and Chl-*a* were set as baseline variables [15]. Spearman’s rank correlation analysis was used to evaluate all 16 environmental factors to address multicollinearity. Predictors with high collinearity (|*r_s_*| > 0.7 and *p* < 0.05) were excluded at this step [32]. Subsequently, multicollinearity among the retained variables was further assessed using variance inflation factor (VIF) diagnostics. A threshold of VIF < 7.5 ruled out severe multicollinearity [33]. All statistical analyses were performed in R v4.3.1. Correlations were computed using the cor.test function and visualized using the “corrplot” (v0.95) and “GGally” (v2.4.0) packages, whereas VIF diagnostics were executed using the “car” package (v3.1–5). The final subset of independent environmental drivers was then utilized to construct GAMs and GLMs for predicting *O. oratoria* biomass.

### 2.5. Comparative Analysis of the GAM and GLM

To identify the optimal functional relationships between the retained environmental drivers and *O. oratoria* biomass, and to mitigate potential bias from model misspecification, GAMs and GLMs were comparatively constructed using Equations (8) and (9), respectively, as follows [19]:

(8) $$\ln(Y\;+\;1)\;=\;\alpha \;+\;\sum_{j\;=\;1}^{n}f_{i}\left(x_{j}\right)\;+\;\varepsilon ,\;\mathrm{and}$$

(9) $$\ln(Y\;+\;1)\;=\;\mu +x_{1\;}+x_{2}+\cdots +x_{j}+\varepsilon ,$$

where *x_j_* represents the explanatory variables, α is the intercept, *ε* denotes the residual error, and *f_i_*(*x_j_*) represents the spline smoothing function. GAMs were fitted to log-transformed biomass using the mgcv package, with a Gaussian distribution, identity link, thin-plate regression splines (default *k* = 10), and GCV.Cp smoothing-parameter estimation. Stepwise AIC selection was used to identify parsimonious models, and the final model was selected by comparing AIC and cumulative deviance explained (CDE) between the GAM and GLM frameworks [34].

### 2.6. HSI Model Construction

The selected GAM was used to construct the habitat suitability model. The partial effects of key environmental drivers on *O. oratoria* biomass during spring and summer were extracted and standardized into a single-factor suitability index (SI) ranging from 0 to 1 using Equation (10) as follows:

(10) $$SI_{k}\left(x\right)\;=\;\frac{f_{k}\left(x\right)\;-\;\min\;[f_{k}(x)]}{\max[f_{k}(x)]\;-\;\min[f_{k}(x)]}$$

where *f_k_*(*x*) represents the fitted partial effect of the k-th environmental predictor over its observed range. This transformation assigned values of 0 and 1 to the minimum and maximum fitted partial effects, respectively. The overall HSI was subsequently calculated as the geometric mean of the individual SI values using Equation (11) [35]:

(11) $${HSI}_{GAM}\;=\;{\left(\prod_{k\;=\;1}^{n}SI_{k}\right)}^{1/n},$$

where *n* represents the number of incorporated environmental variables, and *SI_k_* denotes the suitability index of the *k*-th variable. Station-level HSI values were interpolated in Surfer v16.0 using ordinary kriging with a linear variogram, all 50 stations, and 100 nodes along the longer grid dimension; the resulting surface was clipped to the study-area boundary. For descriptive mapping, HSI values were classified as low (<0.3), moderate (0.3–0.7), and high (≥0.7). These classes represent internally fitted, exploratory suitability rather than independently validated predictions or biological thresholds.

### 2.7. Statistical Analyses

The normality and homogeneity of variance for body length, body weight, and biomass data were verified using the Kolmogorov–Smirnov and Levene’s tests, respectively, to ensure the assumptions of the analysis of variance (ANOVA) were met. Statistical results are expressed as mean ± standard error. The station–season tow was treated as the inferential unit; individual body length and body weight were averaged within each station–season tow, and station identity was specified as the repeated subject in analyses combining the two seasons. One-way ANOVA was used to assess the main effects of habitat grade or season on these indices, whereas two-way ANOVA was utilized to evaluate the interactive effects of habitat grade and season on these indices. Post hoc multiple comparisons were performed using Tukey’s honestly significant difference test, with statistical significance considered at *p* < 0.05. All statistical procedures were executed using IBM SPSS Statistics v22.0 (IBM Corp., Armonk, NY, USA).

## 3. Results

### 3.1. Growth Parameters, Mortality Parameters, and Exploitation Rate of O. oratoria

**Figure 2:**
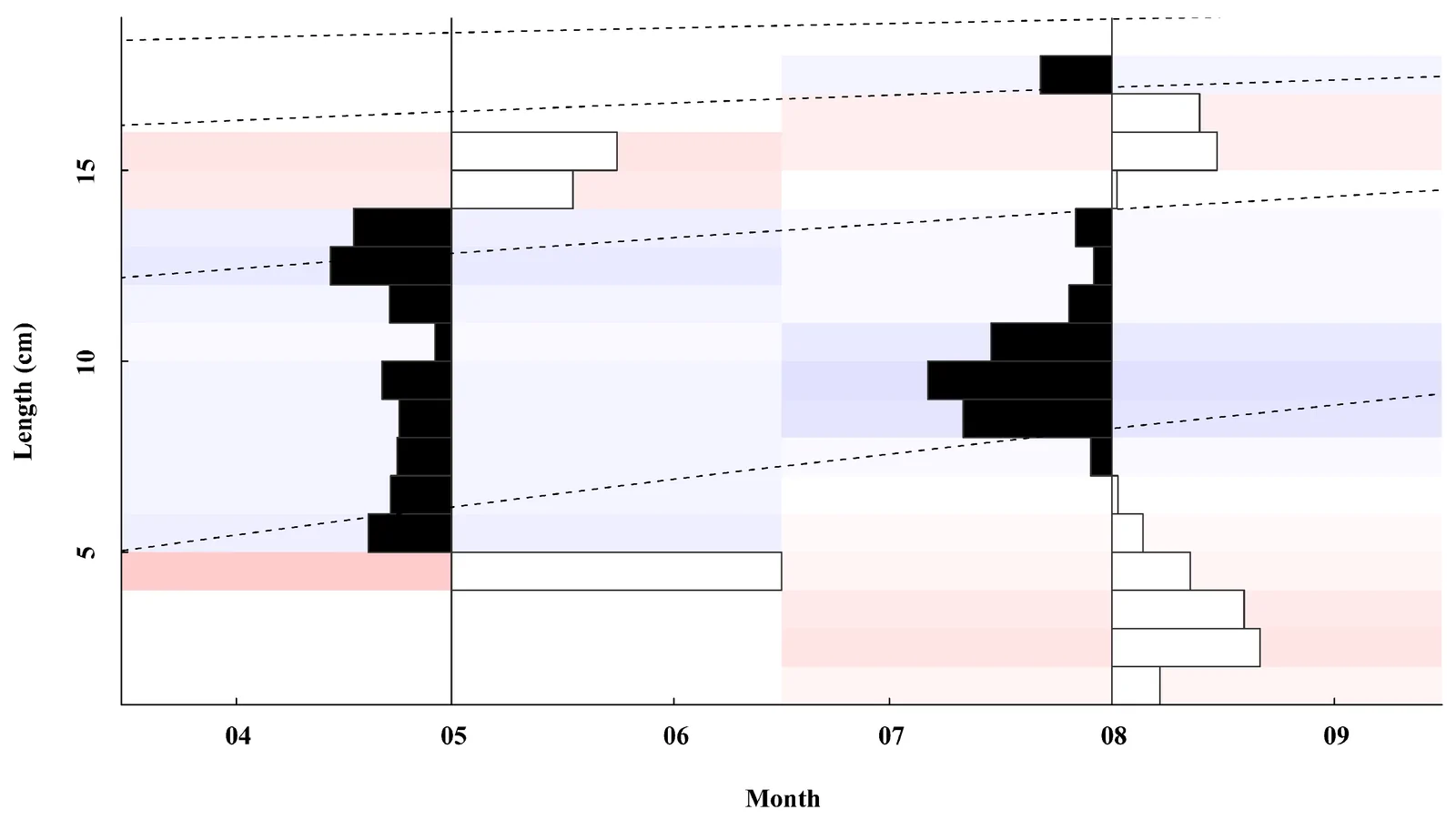
Length–frequency distributions observed in May and August 2024 and ELEFAN-fitted seasonal growth curves of *Oratosquilla oratoria* Black and white bars indicate positive and negative peaks in the restructured data, respectively, and dotted lines represent the fitted growth curves. Months without histograms indicate model-derived trajectories rather than additional sampling.

The length–weight relationship of *O. oratoria* was *W* = 0.04 *L*^2.57^, indicating negative allometric growth (Figure S1). The allometric growth coefficient (*b* = 2.57) indicated a negative allometric growth pattern (*b* < 3). The seasonal growth model estimated *L*_∞_ = 21.20 cm, *K* = 0.58 a^−1^, *t*_0_ = −0.30 a, *C* = 0.76 and *t*_s_ = 0.46, with an ELEFAN fitness score of *R_n_* = 0.346; the fitted curve indicated accelerated growth around June (Figure 2). The catch curve yielded Z = 2.71 a^−1^, while *M*, *F*, and *E* were estimated at 1.27 a^−1^, 1.44 a^−1^, and 0.53, respectively. The length at first capture (*L*_c_) derived from the logistic selectivity curve was 6.48 cm (Figure S3). Accordingly, the biological reference points for optimal length (*L*_opt_) and optimal length at first capture (*L*_copt_) were calculated as 12.26 cm and 10.34 cm, respectively.

### 3.2. Population Dynamics Characteristics of O. oratoria

**Figure 3:**
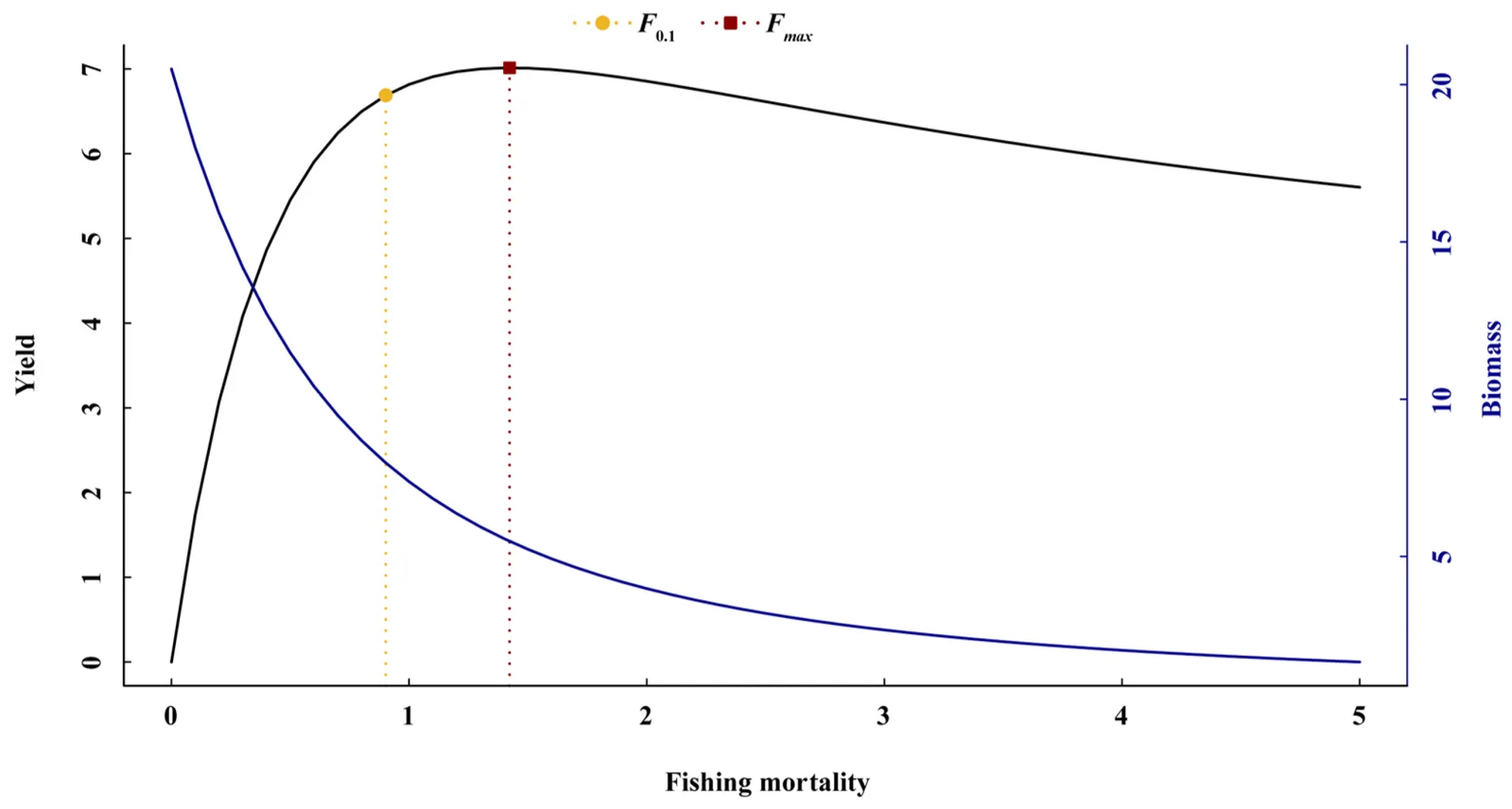
Response curves of yield-per-recruit (YPR) and biomass-per-recruit (BPR) for *Oratosquilla oratoria* as a function of fishing mortality (*F*). The yellow and purple dashed lines represent *F*_0.1_ and *F_max_*, respectively. The black solid curve represents Y/R, whereas the blue solid curve represents B/R.

In the YPR and BPR models, the *Y/R* initially increased rapidly before gradually declining with rising fishing mortality (*F*), reaching its theoretical maximum at *F_max_*. Conversely, *B/R* followed a continuous downward trend as *F* increased, although the rate of decline decelerated at relatively higher *F* levels (Figure 3). Key YPR parameters included *F*_0.1_ = 0.90 a^−1^ (*YPR*_0.1_ = 6.69) and *F_max_* = 1.56 a^−1^ (*YPR_max_* = 7.01). Correspondingly, the BPR model yielded *F*_20%_ = 1.46 a^−1^ (*BPR*_0.2_ = 3.74) and *F*_40%_ = 0.71 a^−1^ (*BPR*_0.4_ = 7.49). Current *F* exceeded *F*_0.1_, indicating growth overfishing, and was close to *F*_20%_.

**Figure 4:**
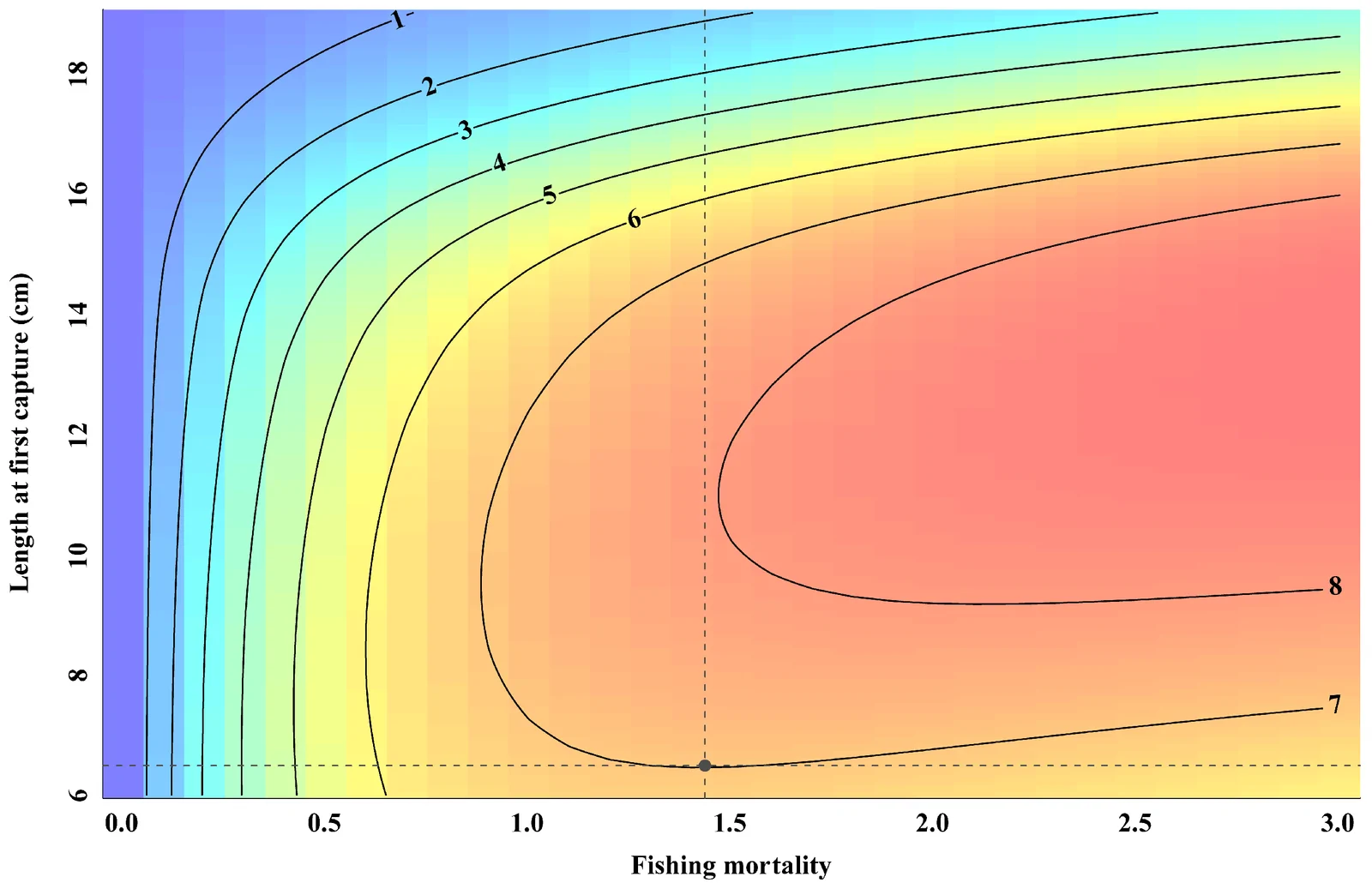
Yield-per-recruit (YPR) isopleths for *Oratosquilla oratoria* in the northern Shandong Peninsula (2024). Colors from blue to red indicate increasing YPR. The black dot denotes the current exploitation status.

The *Y/R* isopleth diagram showed the combined effects of *L*_c_ and *F* on relative yield (Figure 4). The point representing the current combination of *F* and *L*_c_ corresponded to a relatively high *Y/R* but lay outside the peak-yield region. Current *L*_c_ was substantially lower than both *L*_opt_ and *L*_copt,_ indicating premature capture of small individuals. Increasing *L*_c_ toward *L*_copt_ may therefore help conserve population biomass while maintaining long-term fishery yield. The 30-run stability analysis consistently supported *F* > *F*_0.1_ and *L*_c_ < *L*_copt_, whereas the overlapping ranges of *F* and *F*_20%_ indicated that the precise position of current *F* relative to *F*_20%_ was sensitive to parameter variation (Table S2).

### 3.3. Screening Results for Habitat Environmental Factors

**Figure 5:**
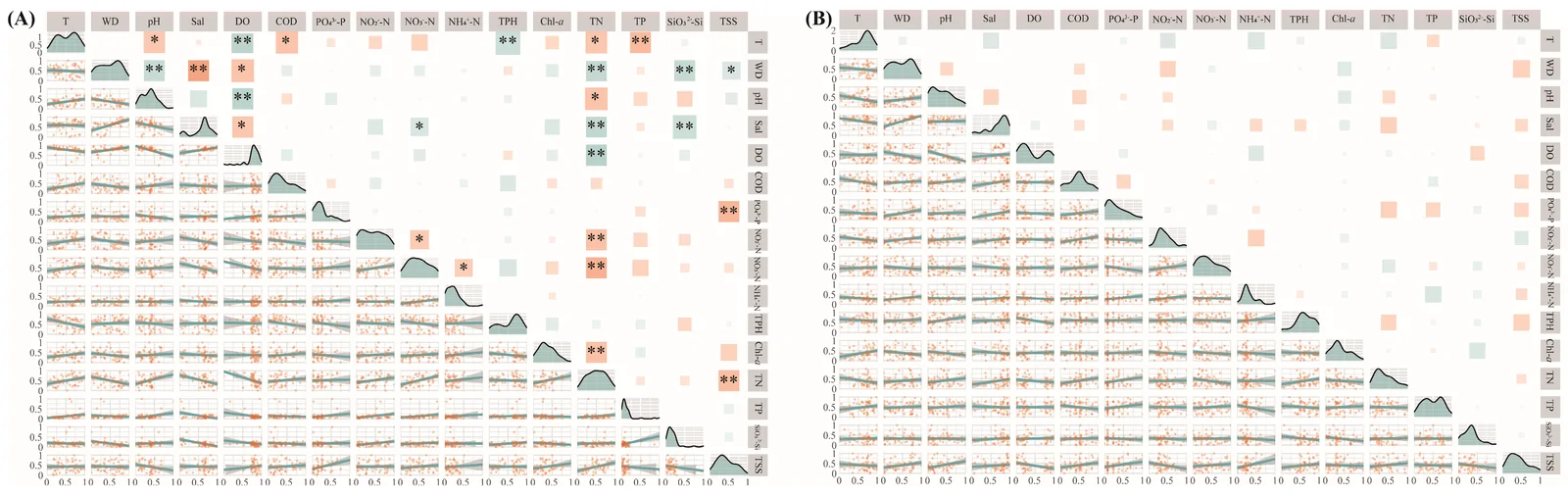
Interrelationships among environmental variables in (**A**) spring and (**B**) summer. The upper triangular section presents Spearman correlation coefficients, with statistical significance indicated by asterisks (*: *p* < 0.05; **: *p* < 0.01). Positive correlations are rendered in orange gradients, while blue gradients represent negative associations. The lower triangular portion contains scatter plots characterizing pairwise relationships between factors. The diagonal panels show the distributions of individual environmental variables.

Diagonal kernel density and scatter plots revealed a wide, non-linear distribution of environmental data, deviating from parametric assumptions and justifying the use of Spearman’s rank correlation analysis (Figure 5). In spring, COD, TN (*p* < 0.05), and TP (*p* < 0.01) were excluded owing to significant positive correlations with T, whereas TPH (*p* < 0.01) was removed for its negative correlation with T. Additionally, SiO_3_^2−^-Si (*p* < 0.01) and TSS (*p* < 0.05) were omitted for their negative correlations with WD, and NO_3_^−^-N (*p* < 0.05) with Sal. In summer, PO_4_^3−^-P (*p* < 0.01) and NH_4_^+^-N (*p* < 0.05) were removed for correlating with WD, TSS (*p* < 0.01) and TPH (*p* < 0.05) with pH, and NO_3_^−^-N (*p* < 0.05) with DO. Ultimately, nine variables were retained for spring (T, Sal, WD, pH, DO, Chl-*a*, NO_2_^−^-N, NH_4_^+^-N, and PO_4_^3−^-P) and summer (T, Sal, WD, pH, DO, Chl-*a*, COD, SiO_3_^2−^-Si, and TP). All VIFs remained below 7.5, ensuring the statistical robustness for subsequent modeling (Figure S4).

### 3.4. Results of the Comparative Analysis of the GAM and GLM Models

**Figure 6:**
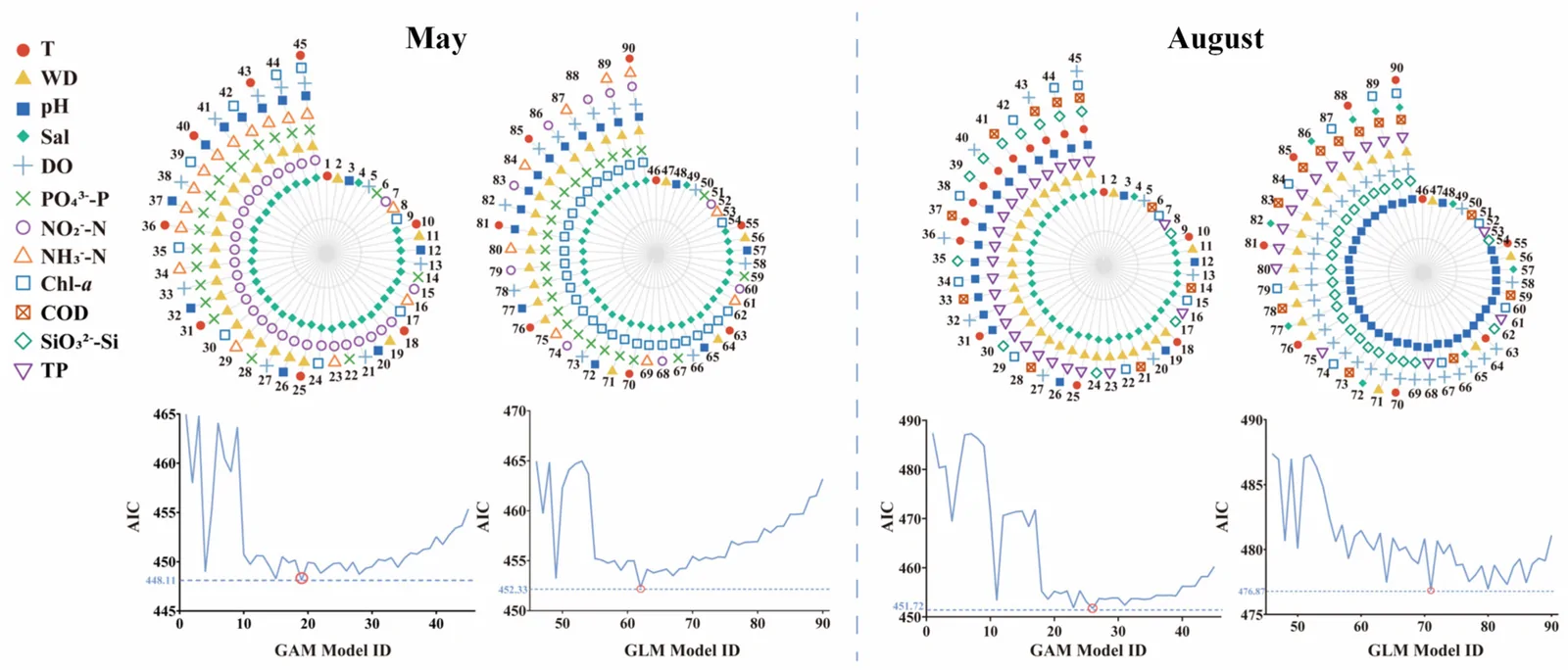
Stepwise selection of environmental drivers for *Oratosquilla oratoria* biomass in spring and summer. Red circles denote the minimum AIC values of the optimal GAM and GLM configurations.

During model selection, GAMs consistently outperformed GLMs (Figure 6). For spring, the optimal GAM configuration (GAM 19: Sal + NO2^−^-N + WD) yielded a lower AIC (448.11) and a higher CDE (43.08%) than the optimal GLM (AIC = 452.33; CDE = 25.38%). Similarly, for summer, the optimal GAM configuration (GAM 26: Sal + WD + TP + pH) exhibited a superior fit (AIC: 451.72; CDE: 57.67%) compared with that of its GLM counterpart (AIC: 476.87; CDE: 28.29%). Consequently, these GAMs were utilized to analyze the partial effects of environmental factors on *O. oratoria* biomass.

**Figure 7:**
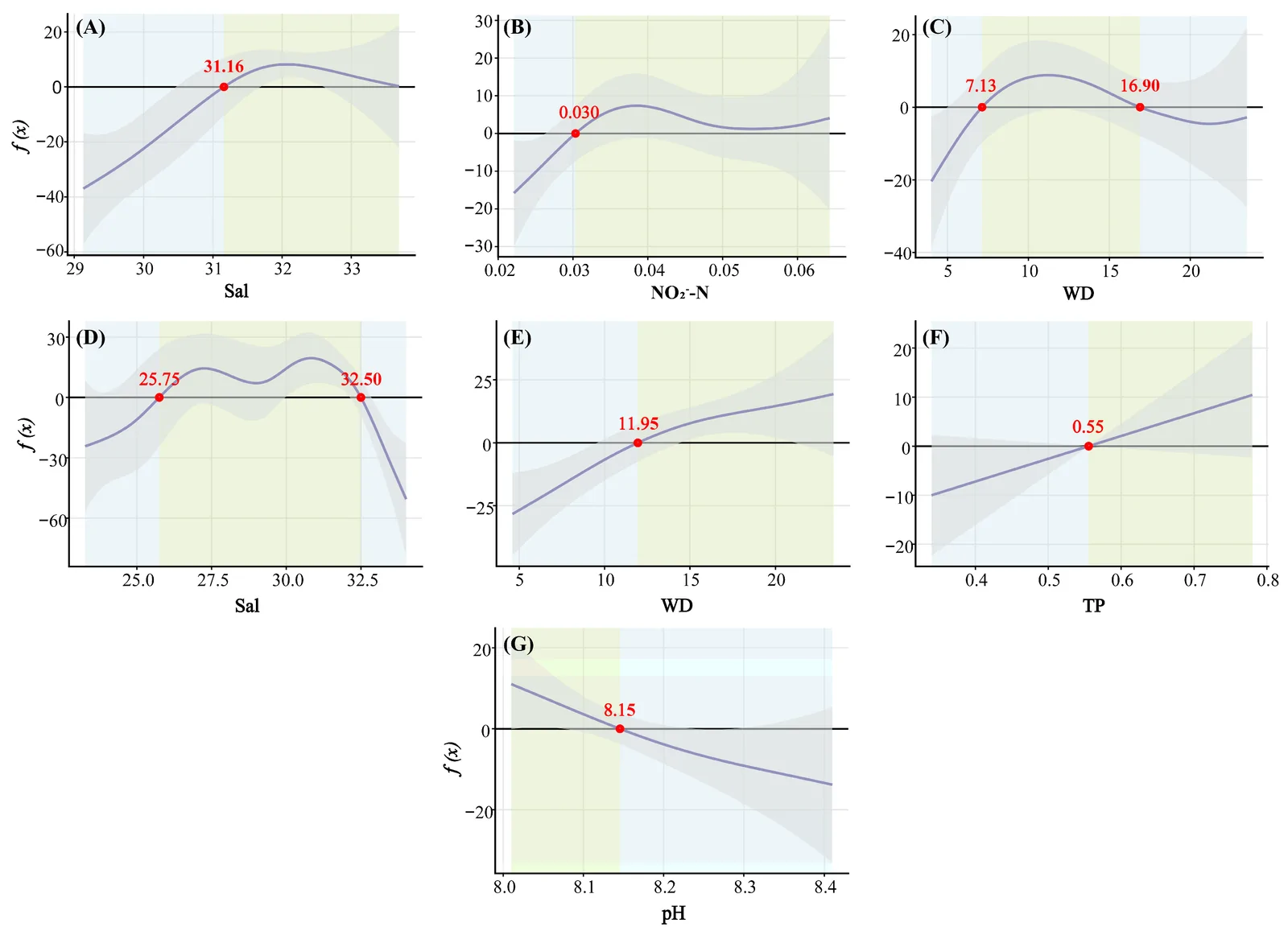
Partial effects of environmental variables on the biomass of *Oratosquilla oratoria* estimated using generalized additive models (GAMs). Panels (**A**–**C**) show the fitted effects of salinity (Sal), nitrite nitrogen (NO_2_^−^-N), and water depth (WD), respectively, in spring, whereas panels (**D**–**G**) show those of salinity (Sal), water depth (WD), total phosphorus (TP), and pH, respectively, in summer. Purple lines represent the estimated smooth effects, and gray shaded areas indicate 95% confidence intervals. Green and blue backgrounds indicate regions with positive and negative fitted effects, respectively, relative to the zero-effect reference line (black horizontal line). Red points indicate selected zero crossings or local turning points of the fitted curves for model interpretation.

In spring, environmental ranges for Sal, WD, and NO_2_^−^-N were 29.13–33.69‰, 4.0–23.5 m, and 0.022–0.064 mg·L^−1^, respectively, with all three variables exhibiting unimodal partial effect curves (Figure 7). Specifically, Sal exhibited positive partial effects above 31.16‰ (positive partial-effect range: 31.16–33.69‰), WD was most favorable between 7.13 and 16.90 m (peaking at ~11.5 m), and NO_2_^−^-N reached its maximum contribution at 0.038 mg·L^−1^ before diminishing. Conversely, in summer, partial effects across the ranges of 23.28–34.01‰ (Sal), 4.60–23.40 m (WD), 0.29–0.78 mg·L^−1^ (TP), and 8.01–8.41 (pH) displayed distinct patterns. Sal followed a multi-modal wavy trend (positive partial effects between 25.75 and 32.50‰), whereas WD and TP showed positive partial effects above 11.95 m and 0.55 mg·L^−1^, respectively, and pH showed negative partial effects above 8.15.

### 3.5. Habitat Suitability Classification and Spatial Distribution Characteristics of O. oratoria

**Figure 8:**
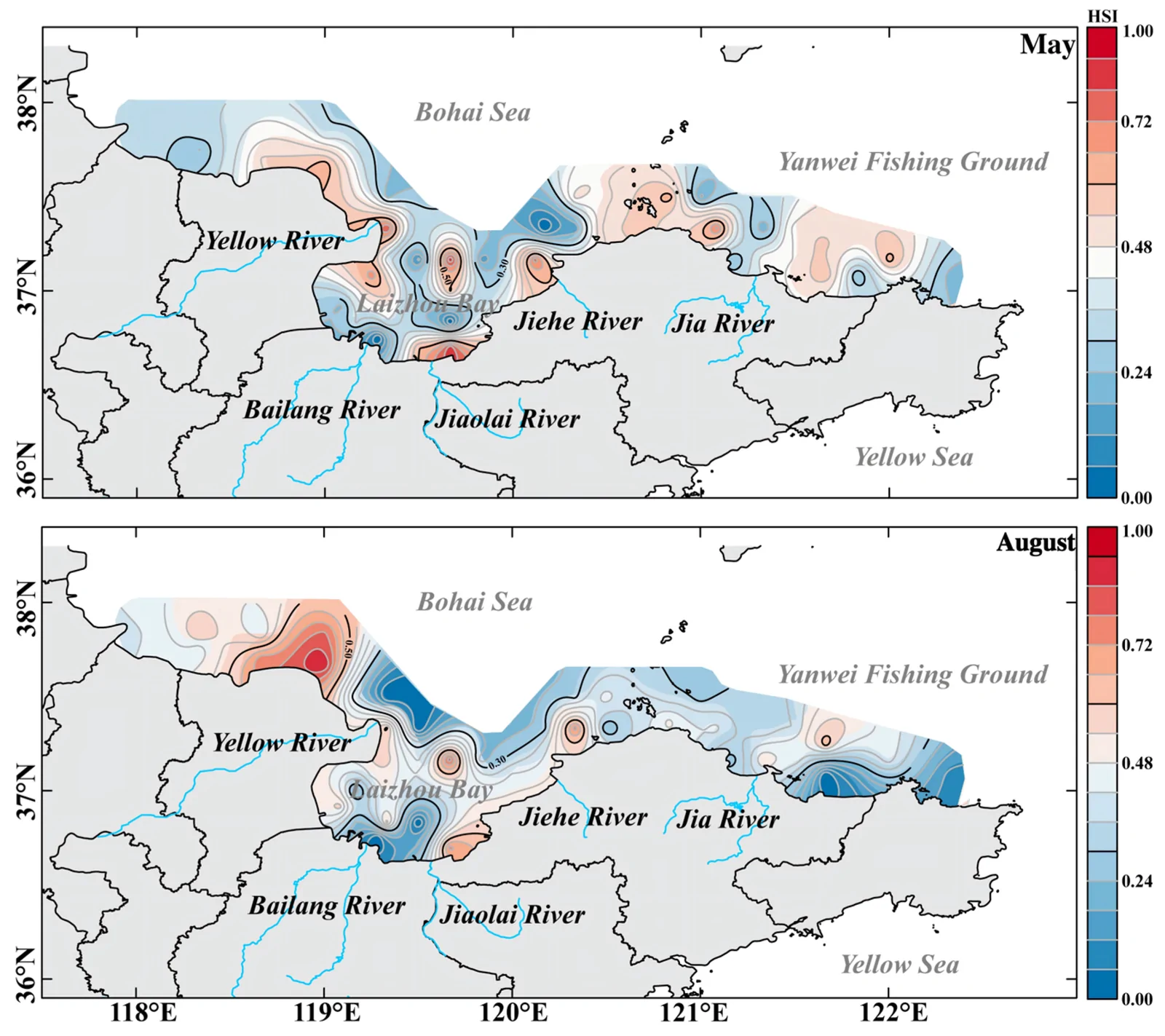
Spatial distribution of model-derived habitat suitability for *Oratosquilla oratoria* in the coastal waters of the northern Shandong Peninsula, China. The color bar represents the gradient of HSI values from 0 to 1.

The model-derived HSI for *O. oratoria* showed seasonal spatial variation (Figure 8). In spring, HSI_GAM_ values ranged from 0 to 0.83 (mean: 0.41), with stations categorized into high (12.0%, *n* = 6), moderate (54.0%, *n* = 27), and low (34.0%, *n* = 17) habitat suitability. Notably, the moderate suitability areas predominated, whereas the high suitability areas were the smallest and most fragmented. Summer HSI_GAM_ ranged from 0 to 0.90 (mean: 0.40), maintaining identical class proportions to spring. Spatially, spring high suitability (HSI_GAM_ ≥ 0.7) was concentrated in northern Laizhou Bay (A24, 0.83), the Jiaolai River Estuary (A27, 0.82), the Yellow River Estuary (A10, 0.78; A15, 0.70), the Jiehe River Estuary (A31, 0.75), and eastern Penglai (B04, 0.73). Moderate suitability areas (0.3 ≤ HSI_GAM_ < 0.7) spanned the western Yellow River Estuary, western Laizhou Bay, and the Yanwei Fishing Ground. In summer, high suitability areas persisted in northern Laizhou Bay (A24, 0.81) and the Jiaolai Estuary (A27, 0.73), whereas new centers emerged in the western Yellow River Estuary (A07, 0.90) and western Penglai (A34, 0.75). Moderate suitability areas showed a broader mapped distribution in parts of the Yellow River Estuary and Yanwei Fishing Ground in summer, although the station-based class proportions were unchanged between seasons.

### 3.6. Differences in the Biological and Population Dynamics Characteristics of O. oratoria Across Habitat Suitability Grades

The two-way ANOVA results revealed the significant effects of the interactions between season and habitat suitability grade on the biomass, body length, and body weight of *O. oratoria* (*p* < 0.05), whereas the interactive effects on abundance were not significant (*p* = 0.715; Table S3). Specifically, spring biomass significantly declined with decreasing habitat suitability, with high suitability areas (1.82 kg·km^−2^) yielding significantly higher biomass than in low suitability areas (0.52 kg·km^−2^; *p* < 0.05). In contrast, summer biomass exhibited no significant differences among habitat suitability grades (0.98–1.27 kg·km^−2^; *p* > 0.05). Seasonally, biomass in low suitability areas was significantly higher in summer (1.27 kg·km^−2^) than in spring (0.52 kg·km^−2^; *p* < 0.05), whereas other suitability grades remained seasonally stable (*p* > 0.05; Table S4). Although a significant main effect of season on abundance was detected (*p* < 0.05; Table S3), post hoc comparisons revealed no significant pairwise differences across seasons or habitats (*p* > 0.05; Table S4). Body length and weight exhibited a significant decrease with declining habitat suitability in spring, whereas both indices were significantly higher in low-suitability areas than in high- and moderate-suitability areas in summer (*p* < 0.05). Moreover, body length and weight in high and moderate suitability areas were significantly greater in spring than in summer, whereas the opposite was true in low suitability areas (*p* < 0.05).

Other population dynamics parameters across habitat suitability grades are summarized in Table S5. Although variations in the length–weight scaling coefficient (*a*) were minimal, the allometric coefficient (*b*) was notably higher in high and moderate suitability areas (2.66) than in the population mean (2.57) and low-suitability areas (2.51). Both the growth coefficient (*K*) and asymptotic length (*L*_∞_) followed a consistent spatial hierarchy of low > high > total > moderate. Estimated *t*_0_ ranged from −0.33 to −0.19 a. Total mortality rate (*Z*) and exploitation rate (*E*) exhibited similar spatial trends (low < moderate < high); however, fishing mortality rate (*F*) decreased as habitat suitability declined, whereas natural mortality rate (*M*) was minimized in moderate suitability areas (moderate < high < low). Furthermore, the length at first capture (*L*_c_) decreased in tandem with habitat suitability, whereas *L*_opt_ and *L*_copt_ mirrored the spatial patterns of *K* and *L*_∞_ (low > high > total > moderate).

## 4. Discussion

### 4.1. Population Dynamics and Exploitation Status of O. oratoria

Population parameters derived from biological dynamics models reflect population responses to environmental stress and fishing pressure [8]. *Oratosquilla oratoria* in the northern Shandong Peninsula exhibited a negative allometric growth pattern (*b* < 3), indicating that weight increases at a slower rate than does length [30]. Although this result aligns with certain findings (b = 2.85) [36], it contrasts sharply with the isometric growth observed in Tokyo Bay (b = 3.0) [37] and the positive allometry in the Mediterranean (b = 3.02–3.26) [38,39]. These regional differences may reflect variation in environmental conditions, food availability, population structure, reproductive status, sampled size ranges, and survey periods [22]. Furthermore, the estimated asymptotic length (*L*_∞_ = 21.2 cm) and growth coefficient (*K* = 0.58 a^−1^) were relatively high compared with those reported for other populations of *O. oratoria* [36,37]. These differences in fitted growth parameters may reflect variation in environmental conditions, food availability, population structure, season, size composition, or methodological differences among studies; however, the present study does not distinguish among these possible influences. The ELEFAN-soVBGF model indicated accelerated growth around June. This model-derived timing broadly coincides with the early reproductive period reported for *O. oratoria* in other regions [36,40]. Beyond growth dynamics, the estimated total mortality rate (*Z* = 2.71 a^−1^) was near the upper end of the range reported in previous crustacean studies (0.86–3.10 a^−1^) [37,39]. Concurrently, the overall exploitation rate (*E* = 0.53) slightly exceeded the commonly used reference level of 0.5 [41], with fishing mortality (*F* = 1.44 a^−1^) contributing slightly more than natural mortality (*M* = 1.27 a^−1^) to total mortality. Compounding this issue, the current length at first capture (*L*_c_ = 6.48 cm) is substantially lower than our estimated biological reference points of *L*_opt_ and *L*_copt_, as well as historical data [42,43]. The combination of relatively high fishing mortality and a small *L*_c_ therefore increases the removal of small individuals from the population. The use of a single bottom trawl with a 20 mm cod-end may have underrepresented small individuals and those in non-trawlable habitats [43]. Therefore, the estimates mainly represent the trawl-accessible component of the population.

The *O. oratoria* stock was subjected to substantial fishing pressure, suggesting that fishing mortality should be reduced to support its conservation and rational exploitation [36]. For instance, reducing the current fishing mortality from 1.44 a^−1^ to *F*_0.1_ = 0.90 a^−1^ would represent a reduction of approximately 37.5%, while retaining most of the predicted yield per recruit. As *F* increased from *F*_40%_ (0.71 a^−1^) to *F*_20%_ (1.46 a^−1^), BPR effectively declined by approximately 50%, from 7.49 to 3.74, indicating a substantial reduction in the biomass retained from each recruit under increasing fishing pressure. The size selectivity of current fishing gears further compounds this issue. The typical length at first sexual maturity for *O. oratoria* ranges from 7 to 8 cm [40], whereas the estimated *L*_c_ (6.48 cm) was below this range. Together with *L*_c_ < *L*_copt_, this result indicates premature capture of small individuals and suggests that some individuals may be removed before contributing fully to cohort biomass and reproduction. Increasing *L*_c_ toward *L*_copt_ may therefore help conserve population biomass and maintain long-term fishery yield [28]. The 30-run stability analysis consistently showed *F* > *F*_0.1_ and *L*_c_ < *L*_copt_ (Table S2).

### 4.2. Environmental Drivers and Habitat Suitability of O. oratoria

The selection of environmental drivers forms the foundation of species distribution models, dictating their reliability in elucidating population dynamics [32]. Extensive research confirms that specific baseline variables are intrinsically linked to the ontogeny and population-level stress responses of *O. oratoria* [18,44,45]. For instance, it has been reported that temperature and Sal directly govern metabolic rates and osmoregulatory capacity, underpinning the species’ eurythermal and euryhaline adaptations. Concurrently, WD dictates benthic pressure gradients and vertical light attenuation, directly impacting behavioral and spatial distributions. DO and pH emerge as critical limiting factors amid global climate change and coastal eutrophication, with hypoxia and ocean acidification severely restricting survival and recruitment [7]. Moreover, Chl-*a* concentrations, a proxy for primary productivity, directly reflect the potential availability of prey [46]. Consequently, these established baseline variables were incorporated directly into the population dynamics models without prior screening. Furthermore, the seasonally retained model variables highlight the complexity of the drivers modulating population fluctuations. Specifically, elevations in NH_4_^+^-N and PO_4_^3−^-P levels during spring promote phytoplankton blooms, providing abundant nutritional resources for broodstock maturation and larval development [46]; however, concurrent peaks in NO_2_^−^-N levels highlight the potential toxicological risks of nitrogen-cycle intermediates to spawning adults [47]. In summer, elevated COD and TP levels reflect the influx of terrestrial runoff and precipitation, signaling the heightened risks of eutrophication and organic pollution that severely impair *O. oratoria* health during its peak metabolic and developmental phases. Additionally, fluctuations in SiO_3_^2−^-Si concentrations can alter diatom community structures, directly impacting the availability of high-quality prey [48].

GAMs and GLMs are routinely employed to evaluate functional relationships between organisms and environmental drivers [19]. The GAM framework, owing to its capacity to capture complex, non-linear ecological responses, was selected because it provided a better in-sample fit than the GLM framework [16]. In spring, Sal showed the strongest modeled association with biomass, corroborating Li et al. [6] and Zhang et al. [15]. The fitted partial effect of Sal became positive above 31.16‰ and remained positive between 31.16 and 33.69‰, which may reflect relatively favorable osmoregulatory conditions within the observed range [18]. With only 10 stations falling outside this positive modeled range, Sal generally showed a positive modeled association with biomass. Conversely, spring NO_2_^−^-N concentrations (0.022–0.064 mg·L^−1^) were lower than previously reported (0.0745 ± 0.05 mg·L^−1^) [49]. Although insufficient NO_2_^−^-N (<0.030 mg·L^−1^) restricts prey availability, rapid elevations (>0.038 mg·L^−1^) induce toxicity and impair growth [47], highlighting the acute sensitivity of the population to fluctuations in NO_2_^−^-N levels. Furthermore, the positive partial-effect range for WD in spring (7.13–16.90 m) corroborates the “overwintering in deep water, spawning in shallows” migratory behavior of this species [6]. However, over half of the surveyed stations (26) fell outside this positive modeled range, suggesting relatively less favorable modeled conditions at these stations. During summer, Sal remained an important predictor, with positive partial effects between 25.75 and 32.50‰. The narrower positive modeled range in summer may be related to seasonal changes in temperature and the energetic costs of osmoregulation [44]. Nevertheless, most stations (36) remained within this positive modeled Sal range. Additionally, biomass showed a positive modeled association with WD above 11.95 m in summer, which may be related to the greater hydrological stability of deeper waters [6]. However, as observed in spring, most summer stations (28) lay outside this positive modeled WD range, indicating relatively lower model-derived suitability with respect to WD. Nutritionally, summer TP concentrations (0.29–0.78 mg·L^−1^) were relatively low compared with those in other regions (0.058–4.62 mg·L^−1^) [50]. Twenty-four stations fell below the model-relative zero crossing of approximately 0.55 mg·L^−1^, suggesting a negative modeled association between TP and biomass below this value. Finally, summer pH values ranged from 8.01 to 8.41, and the fitted partial effect of pH became negative above approximately 8.15. Therefore, with 24 stations situated within the inhibitory, higher-pH range, the ecological risk posed by elevated seawater pH on *O. oratoria* survival warrants serious attention [51]. However, out-of-sample model performance and residual spatial dependence were not assessed, which should be considered when interpreting the model-derived HSI.

### 4.3. Population Variation Among Habitat-Suitability Grades and Spatial Management Implications

The similar station-level HSI values and suitability-class proportions between seasons suggested relatively limited seasonal variation in model-derived habitat suitability [17,18]. However, the spatial landscape, dominated by moderate suitability areas alongside highly fragmented, patchy high and low suitability areas, indicates pronounced spatial heterogeneity in model-derived habitat suitability. Furthermore, these suitable habitats exhibited seasonal spatial redistribution. The high suitability areas in spring were heavily concentrated in estuarine habitats, such as the Jiaolai, Yellow, and Jiehe River Estuaries, broadly consistent with the species’ reported use of inshore shallow waters for spawning and early foraging [6]. In summer, these areas persisted, with new high suitability centers emerging west of the Yellow River Estuary and west of Penglai. The former may provide some buffering against salinity fluctuations and increased turbidity associated with summer runoff [52], whereas the robust hydrodynamic exchange in the latter may reduce the likelihood of stratification-induced hypoxia [53]. The broader mapped distribution of moderate-suitability areas in summer may reflect seasonal changes in the modeled environmental associations. Conversely, low-suitability patches showed limited seasonal redistribution, which may reflect relatively unfavorable combinations of the modeled environmental variables [54]. Crucially, this spatial heterogeneity of model-derived habitat suitability may be associated with variation in population distribution, structure, and dynamics [15]. In spring, *O. oratoria* showed higher biomass in high suitability areas, potentially reflecting more favorable environmental conditions [6,55]. Paradoxically, individuals in low suitability areas had significantly greater body length and weight than those in high-suitability areas during summer. This pattern may be consistent with redistribution among habitats [56]. Concurrently, high-suitability areas also exhibited higher estimated fishing mortality, which could contribute to the preferential removal of larger individuals from the population. Ultimately, ecological responses, such as compensatory growth and rapid energy accumulation, could potentially contribute to the larger body size observed in low-suitability areas during summer [57].

The spatial heterogeneity of model-derived habitat suitability was associated not only with the distribution of *O. oratoria* but also with spatial variation in its mortality and exploitation rates [14]. The elevated *Z*, *F*, and *E* values in high suitability areas, juxtaposed with the higher *M* value in low suitability areas, reveal a distinct spatial mismatch between fishing pressure and environmental stress. The coincidence of high model-derived habitat suitability and elevated fishing mortality could potentially create ecological-trap-like conditions, whereas the higher natural mortality estimated in low suitability areas may be associated with less favorable environmental conditions [58]. Consequently, relying on a uniform, “one-size-fits-all” management approach may be less effective across different habitat regions, thereby undermining the comprehensive benefits for the *O. oratoria* population structure and fishery yields [14].

Based on these assessments, we propose a preliminary spatially differentiated management scenario for the *O. oratoria* fishery. Natural resource conservation areas could be considered within high and moderate suitability areas. High suitability areas could be prioritized for conservation, with 11.89 cm considered as a candidate *L*_c_ reference value. Moderate-suitability areas could serve as experimental buffer areas, with 8.63 cm considered as a preliminary *L*_c_ reference value. Furthermore, reductions in fishing effort aimed at moving *E* toward or below 0.5 could be evaluated in high and moderate suitability areas [59]. Low suitability areas contained relatively large individuals during summer, although movement from other habitat classes was not directly demonstrated. Therefore, rather than immediately imposing strict size or effort limits, a seasonal TAC framework could be explored after sufficient catch, effort, and fleet data become available [60]. Subsequent management adjustments should be informed by long-term monitoring and modeling to evaluate population growth, mortality, and exploitation status. This spatially heterogeneous framework, integrating “core conservation, experimental transition, and marginal utilization,” is broadly consistent with spatially differentiated approaches applied in other benthic crustacean fisheries [61]. Ultimately, this strategy may help reduce fishing pressure while supporting the conservation and sustainable exploitation of *O. oratoria*.

## 5. Conclusions

We established an integrated evaluation framework, linking population dynamics projection, habitat assessment, and management strategy formulation, to elucidate the population dynamics and exploitation status of *O. oratoria* in the northern Shandong Peninsula, as well as its habitat suitability and key environmental drivers.

Specifically, the population exhibited negative allometric growth, with accelerated growth around June. Current *F* exceeded *F*_0.1_, and *L*_c_ remained below *L*_copt_, indicating substantial fishing pressure and premature capture of small individuals. GAMs provided a better in-sample fit than GLMs and identified seasonal associations between biomass and environmental variables. Sal was retained as an important predictor in both seasons, whereas WD and NO_2_^−^-N were retained in spring and WD, TP, and pH in summer. Spatially, the model-derived HSI maps were dominated by moderate-suitability areas interspersed with fragmented high- and low-suitability patches. Seasonal spatial redistribution was observed, although the station-based proportions of the three suitability classes remained unchanged between seasons. Higher fishing mortality and exploitation rates were estimated in high-suitability areas, whereas relatively large individuals occurred in low-suitability areas during summer. Based on these spatial heterogeneities, we propose a preliminary spatially differentiated management scenario, in which 11.89 cm and 8.63 cm may be considered *L*_c_ reference values for high and moderate suitability areas, respectively. Reductions in fishing effort aimed at moving *E* toward or below 0.5 could also be evaluated. For low suitability areas, a seasonal TAC framework could be explored after sufficient catch, effort, and fleet data become available. These proposed measures require further validation through longer-term monitoring. Taken together, the results from this study provide an initial scientific basis for the spatial management of *O. oratoria* and may inform assessments of similar benthic fisheries.

## Data Availability

The data that support the findings of this study are available from the corresponding author upon reasonable request.

## Supplementary Materials

The following supporting information can be downloaded at: https://www.mdpi.com/article/10.3390/ani16172709/s1, Figure S1. Length-weight relationship of *Oratosquilla oratoria* in the coastal waters of the northern Shandong Peninsula, China. Individual observations are displayed as scatter plots, and the blue solid line represents the fitted power function. Figure S2. Length-converted catch curve for *Oratosquilla oratoria* in the coastal waters of the northern Shandong Peninsula, China. The solid line represents the linear regression fitted to the descending limb of the curve, from which the total mortality coefficient (*Z*) is estimated. Figure S3. Catch selectivity curve for *Oratosquilla oratoria* in the coastal waters of the northern Shandong Peninsula, China. The red dashed lines indicate the length at 50% selectivity, which defines the length at first capture (*L*_c_ = 6.48 cm). Figure S4. VIF diagnostics for environmental variables. Only predictors with VIF < 7.5 are shown. Table S1. Geographic coordinates of the 50 fixed sampling stations surveyed in spring and summer 2024 in the coastal waters of the northern Shandong Peninsula, China. Table S2. Numerical stability of growth, mortality, selectivity, and per-recruit parameters estimated from 30 independent ELEFAN runs with different random seeds. Table S3. Results of two-way ANOVA analyzing the effects of season, habitat grade, and their interaction on the biomass, abundance, length, and weight of *Oratosquilla oratoria* in the coastal waters of the northern Shandong Peninsula, China. Table S4. Biological parameters of *Oratosquilla oratoria* across different seasons and habitat zones. Table S5. Growth, mortality, and population characteristic parameters of *Oratosquilla oratoria* across different habitat areas.
