# Supplementary material for: Study of the Population Dynamics and Habitat Suitability of Oratosquilla oratoria in the Coastal Waters of Northern Shandong Peninsula, China
Source: Animals (Basel). 2026 Sep 1;16(17):2709. doi: 10.3390/ani16172709 (PMC13565242; doi:10.3390/ani16172709)
Supplement: Supplementary file 1 [file animals-16-02709-s001.zip › animals-4469870-supplementary.pdf]

Supplementary Materials

Study of the Population Dynamics and Habitat Suitability of *Oratosquilla oratoria* in the Coastal Waters of Northern Shandong Peninsula, China

Shengfu Li [1], Xiaoxia Yu [2], Xiang Yu [3], Wenhui Cui [1], Zhaoyu Song [1], Min Li [1], Yiwen Liu [1], Zhenbo Lv [1], Liang Zheng [4,*] and Zhonghua Ren [1,*]

[1] Advanced Institute for Coastal Ecology, Ludong University, Yantai 264025, China
[2] Key Laboratory of Land and Sea Ecological Governance and Systematic Regulation, Ministry of Ecology and Environment, Shandong Academy for Environmental Planning, Jinan 250101, China
[3] Shandong Marine Resource and Environment Research Institute, Yantai 264006, China
[4] Marine and Fishery Integrated Service Center of Haiyang City, Yantai 265100, China
* Correspondence: zhengliang0372@126.com (L.Z.); renzhonghua16@mails.ucas.ac.cn (Z.R.)

Supplementary Figures 1

Figure S1. Length-weight relationship of *Oratosquilla oratoria* in the coastal waters of the northern Shandong Peninsula, China. 1

Figure S2. Length-converted catch curve for *Oratosquilla oratoria* in the coastal waters of the northern Shandong Peninsula, China. 2

Figure S3. Catch selectivity curve for *Oratosquilla oratoria* in the coastal waters of the northern Shandong Peninsula, China. 3

Figure S4.VIF diagnostics for environmental variables. 4

Supplementary Tables 5

Table S1. Geographic coordinates of the 50 fixed sampling stations surveyed in spring and summer 2024 in the coastal waters of the northern Shandong Peninsula, China.. 5

Table S2. Numerical stability of growth, mortality, selectivity, and per-recruit parameters estimated from 30 independent ELEFAN runs with different random seeds. 7

Table S3. Results of two-way ANOVA analyzing the effects of season, habitat grade, and their interaction on the biomass, abundance, length, and weight of *Oratosquilla oratoria* in the coastal waters of the northern Shandong Peninsula, China. 8

Table S4. Biological parameters of *Oratosquilla oratoria* across different seasons and habitat suitability levels.. 9

Table S5. Growth, mortality, and population characteristic parameters of *Oratosquilla oratoria* across different habitat suitability levels. 10

Supplementary Figures

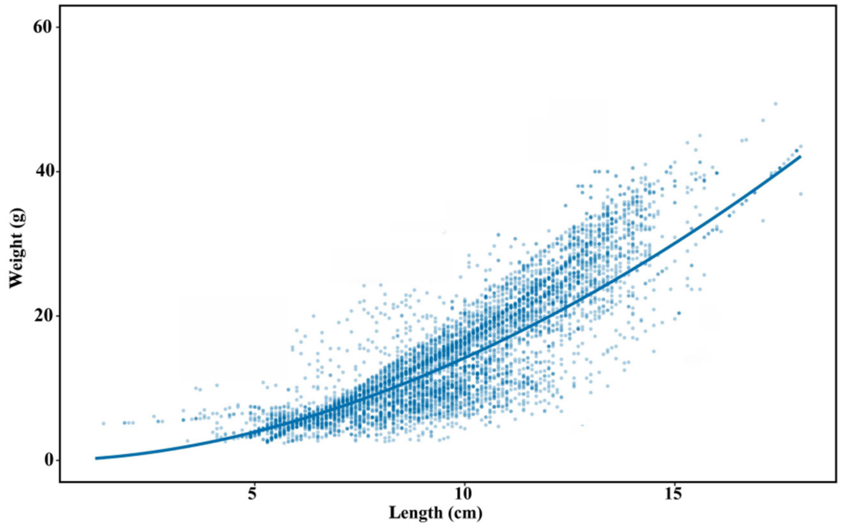

Figure S1. Length-weight relationship of *Oratosquilla oratoria* in the coastal waters of the northern Shandong Peninsula, China. Individual observations are displayed as scatter plots, and the blue solid line represents the fitted power function.

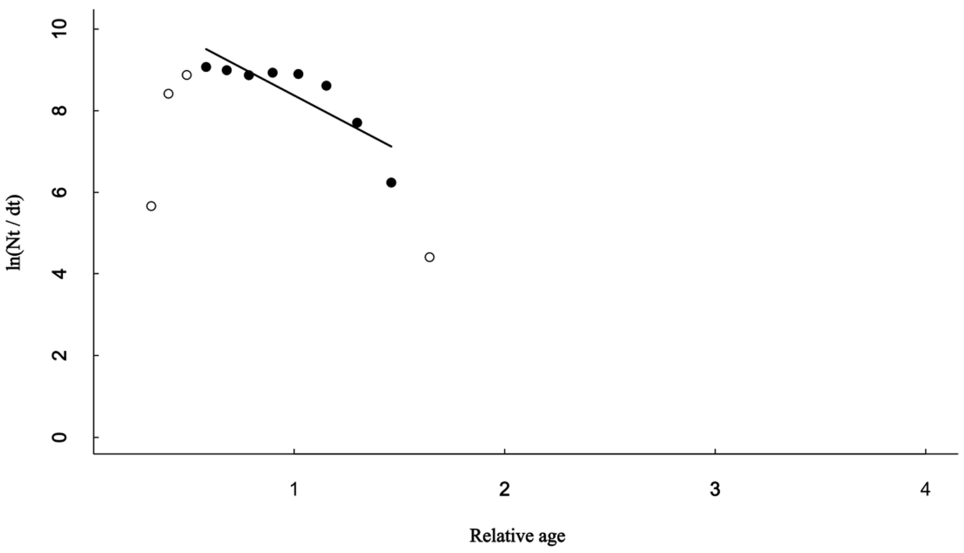

Figure S2. Length-converted catch curve for *Oratosquilla oratoria* in the coastal waters of the northern Shandong Peninsula, China.The solid line represents the linear regression fitted to the descending limb of the curve, from which the total mortality coefficient (*Z*) is estimated.

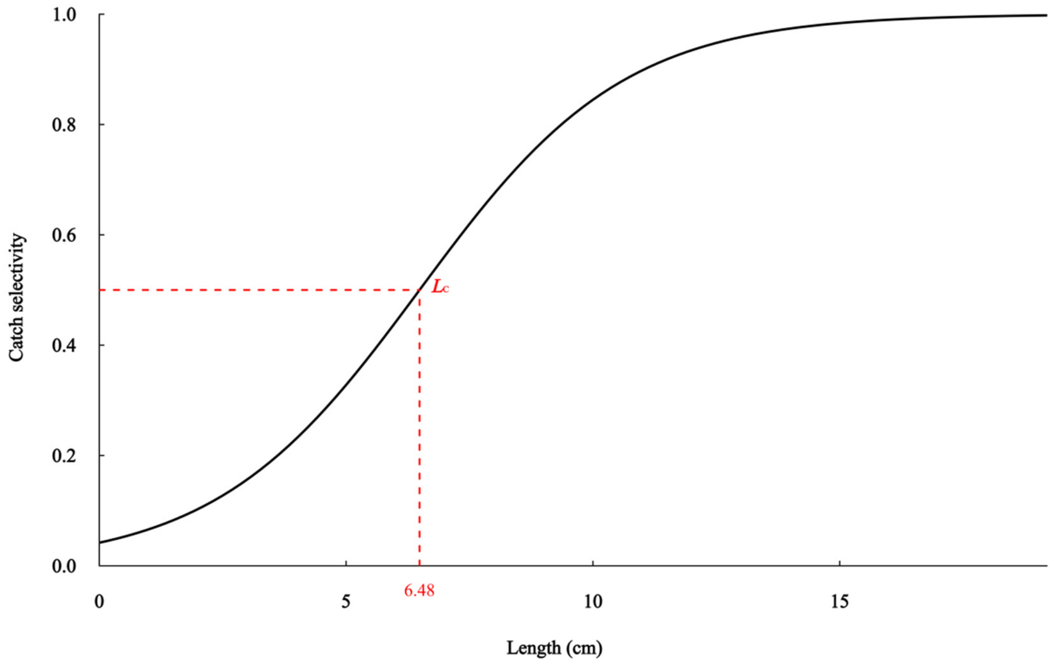

Figure S3. Catch selectivity curve for *Oratosquilla oratoria* in the coastal waters of the northern Shandong Peninsula, China. The red dashed lines indicate the length at 50% selectivity, which defines the length at first capture (L_c = 6.48 cm).

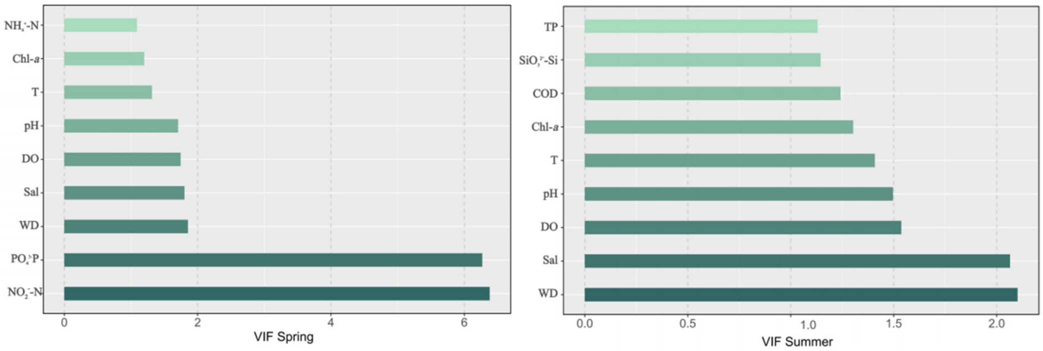

Figure S4. VIF diagnostics for environmental variables. Only predictors with VIF < 7.5 are shown.

Supplementary Tables

Table S1.Geographic coordinates of the 50 fixed sampling stations surveyed in spring and summer 2024 in the coastal waters of the northern Shandong Peninsula, China.

Sampling station	Longitude	Latitude
A01	118°00′00.00″	38°20′00.00″
A02	118°20′00.00″	38°20′00.00″
A03	118°20′00.00″	38°15′00.00″
A04	118°40′00.00″	38°20′00.00″
A05	118°40′00.00″	38°12′00.00″
A06	119°00′00.00″	38°20′00.00″
A07	119°00′00.00″	38°10′00.00″
A08	119°10′00.00″	38°00′00.00″
A09	119°20′00.00″	38°00′00.00″
A10	119°20′00.00″	37°50′00.00″
A11	119°30′00.00″	37°50′00.00″
A12	119°05′00.00″	37°25′00.00″
A13	119°10′00.00″	37°30′00.00″
A14	119°10′00.00″	37°20′00.00″
A15	119°15′00.00″	37°35′00.00″
A16	119°20′00.00″	37°40′00.00″
A17	119°20′00.00″	37°30′00.00″
A18	119°20′00.00″	37°20′00.00″
A19	119°17′00.00″	37°15′00.00″
A20	119°30′00.00″	37°40′00.00″
A21	119°30′00.00″	37°30′00.00″
A22	119°30′00.00″	37°20′00.00″
A23	119°30′00.00″	37°12′00.00″
A24	119°40′00.00″	37°40′00.00″
A25	119°40′00.00″	37°30′00.00″
A26	119°40′00.00″	37°20′00.00″
A27	119°40′00.00″	37°12′00.00″
A28	119°50′00.00″	37°40′00.00″
A29	119°50′00.00″	37°30′00.00″
A30	120°00′00.00″	37°35′00.00″
A31	120°07′00.00″	37°40′00.00″
A32	120°10′00.00″	37°50′00.00″
A33	120°20′00.00″	38°00′00.00″
A34	120°20′00.00″	37°50′00.00″
A35	120°30′00.00″	38°00′00.00″
A36	120°30′00.00″	37°50′00.00″
B01	120°50′00.00″	38°00′00.00″
B02	120°50′00.00″	37°52′00.00″
B03	121°00′00.00″	38°00′00.00″

B04	121°05′00.00″	37°50′00.00″
B05	121°10′00.00″	37°40′00.00″
B06	121°20′00.00″	37°50′00.00″
B07	121°20′00.00″	37°40′00.00″
B08	121°30′00.00″	37°50′00.00″
B09	121°30′00.00″	37°40′00.00″
B10	121°40′00.00″	37°45′00.00″
B11	121°40′00.00″	37°35′00.00″
B12	121°50′00.00″	37°35′00.00″
B13	122°00′00.00″	37°40′00.00″
B14	122°20′00.00″	37°35′00.00″

Table S2. Numerical stability of growth, mortality, selectivity, and per-recruit parameters estimated from 30 independent ELEFAN runs with different random seeds.

**Parameters**	**Reference fit**	**Mean±SD**	**Median (IQR)**	**Minimum–maximum**	**CV (%)**
L_∞	21.2	21.22±0.23	21.23(21.13–21.41)	20.72–21.53	1.06
K	0.58	0.581±0.018	0.580(0.566–0.597)	0.553–0.607	3.06
C	0.76	0.761±0.019	0.762(0.745–0.779)	0.715–0.786	2.53
t_s	0.46	0.458±0.026	0.459(0.446–0.475)	0.405–0.512	5.69
ELEFAN fitness score, *Rn*	0.346	0.338±0.005	0.339(0.336–0.343)	0.325–0.346	1.61
Z	2.71	2.703±0.034	2.701(2.684–2.724)	2.640–2.772	1.24
M	1.27	1.271±0.029	1.267(1.244–1.293)	1.228–1.317	2.24
F	1.44	1.433±0.032	1.430(1.413–1.446)	1.384–1.507	2.2
E	0.531	0.530±0.009	0.531(0.524–0.536)	0.514–0.549	1.73
L_C	6.48	6.49±0.07	6.4 (6.45–6.52)	6.34–6.62	1
L_{opt}	12.26	12.27±0.11	12.29(12.23–12.34)	12.03–12.42	0.9
L_{copt}	10.34	10.35±0.11	10.35(10.30–10.43)	10.09–10.50	1.1
$F_{0.1}$	0.9	0.898±0.017	0.899(0.887–0.906)	0.865–0.934	1.91

$F_{40\%}$	0.71	0.715±0.012	0.714(0.706–0.725)	0.689–0.735	1.71
$F_{20\%}$	1.46	1.461±0.030	1.464(1.438–1.481)	1.405–1.515	2.05
F_{max}	1.56	1.560±0.029	1.564(1.541–1.574)	1.505–1.615	1.86
Model-derived peak-growth period	June	—	June(24/30 runs)	May–July	—

Table S3. Results of two-way ANOVA analyzing the effects of season, habitat grade, and their interaction on the biomass, abundance, length, and weight of *Oratosquilla oratoria* in the coastal waters of the northern Shandong Peninsula, China.

**Factors**	**Biomass ($kg·km^{-2}$)**	**Abundance ($ind.·km^{-2}$)**	**Length (mm)**	**Weight (g)**
	p	***p***	***p***	***p***
Season	0.911	0.014*	<0.001***	<0.001***
Habitat	0.038*	0.447	<0.001***	<0.001***
Season × Habitat	<0.001***	0.715	<0.001***	<0.001***

Note: Significance levels are indicated by asterisks: *p < 0.05 and ***p < 0.001.'Season × Habitat' denotes the interaction effect between the two independent factors. p values are derived from two-way ANOVA analysis.

Table S4. Biological parameters of *Oratosquilla oratoria* across different seasons and habitat zones.

Season	Habitat zones	Biomass (kg·km^{-2})	Abundance (ind.·km^{-2})	Length (mm)	Weight (g)
Spring	high	1.82±0.38^{a}	62.00±10.36^{a}	133.70±0.59^{a*}	29.38±0.34^{a*}
	mid	0.95±0.10^{b}	54.29±4.27^{a}	101.97±0.54^{b*}	17.42±0.19^{b*}
	low	0.52±0.09^{b}	57.44±6.78^{a}	76.32±0.47^{c}	9.12±0.14^{c}
Summer	high	0.99±0.25^{a}	73.17±17.97^{a}	91.97±1.01^{b}	13.53±0.40^{b}
	mid	0.98±0.09^{a}	69.37±5.00^{a}	93.35±0.49^{b}	14.17±0.20^{b}
	low	1.27±0.21^{a*}	81.06±7.64^{a}	97.16±0.56^{a*}	15.64±0.23^{a*}

Note: Data are expressed as mean ± standard error (SE). Different superscript letters within a column for the same season indicate significant differences between habitat zones ($p < 0.05$). Asterisks (*) denote significant differences between seasons within the same habitat zone ($p < 0.05$).

Table S5. Growth, mortality, and population characteristic parameters of *Oratosquilla oratoria* across different habitat areas.

Parameters	Total habitat	High area	Moderate area	Low area
a	0.04	0.02	0.03	0.04
b	2.57	2.66	2.66	2.51
K	0.58 a^{-1}	0.74 a^{-1}	0.56 a^{-1}	0.86 a^{-1}
t_0	-0.30	-0.23	-0.33	-0.19
L_∞	21.2 cm	22.85 cm	17.74 cm	24.08 cm
Z	2.71 a^{-1}	2.94 a^{-1}	2.53 a^{-1}	2.41 a^{-1}
M	1.27 a^{-1}	1.38 a^{-1}	1.22 a^{-1}	1.49 a^{-1}
F	1.44 a^{-1}	1.56 a^{-1}	1.31 a^{-1}	0.92 a^{-1}
E	0.53	0.53	0.52	0.38
L_C	6.48 cm	9.79 cm	8.04 cm	6.15 cm
L_{opt}	12.26cm	14.09 cm	10.28 cm	15.26 cm
L_{copt}	10.34 cm	11.89 cm	8.63 cm	12.12 cm
